# Does Notch play a tumor suppressor role across diverse squamous cell carcinomas?

**DOI:** 10.1002/cam4.731

**Published:** 2016-05-26

**Authors:** Min Zhang, Sangita Biswas, Xin Qin, Wenrong Gong, Wenbing Deng, Hongjun Yu

**Affiliations:** ^1^ Medical CollegeHubei University of Arts and ScienceXiangyangHubeiChina; ^2^ Department of Biochemistry and Molecular MedicineSchool of MedicineUniversity of CaliforniaDavisCalifornia; ^3^ Department of BiologyBrookhaven National LabNewYork

**Keywords:** mutation pattern, Notch pathway, Notch structure, squamous cell carcinoma, tumor suppressor

## Abstract

The role of Notch pathway in tumorigenesis is highly variable. It can be tumor suppressive or pro‐oncogenic, typically depending on the cellular context. Squamous cell carcinoma (SCC) is a cancer of the squamous cell, which can occur in diverse human tissues. SCCs are one of the most frequent human malignancies for which the pathologic mechanisms remain elusive. Recent genomic analysis of diverse SCCs identified marked levels of mutations in *NOTCH1*, implicating Notch signaling pathways in the pathogenesis of SCCs. In this review, evidences highlighting NOTCH's role in different types of SCCs are summarized. Moreover, based on accumulating structural information of the NOTCH receptor, the functional consequences of *NOTCH1* gene mutations identified from diverse SCCs are analyzed, emphasizing loss of function of Notch in these cancers. Finally, we discuss the convergent view on an intriguing possibility that Notch may function as tumor suppressor in SCCs across different tissues. These mechanistic insights into Notch signaling pathways will help to guide the research of SCCs and development of therapeutic strategies for these cancers.

## Introduction

Squamous cell carcinoma (SCC) is a type of cancer caused by uncontrolled proliferation of squamous cells. SCCs are one of the most prevalent malignancies, arising in diverse body sites such as skin, mouth, esophagus, lung, and cervix. Of them, head and neck SCC (HNSCC) is the sixth leading malignancy globally 1, whereas cutaneous SCC (CSCC) is the second most common skin cancer worldwide 2.

Notch homolog 1, translocation associated (Drosophila), also known as *NOTCH1*, is a human gene encoding a single‐pass transmembrane receptor. Notch signaling pathways play a key role in development and tissue homeostasis, and its dysfunction is involved in tumorigenesis and other human diseases 3. However, Notch signaling activation can be tumor suppressive or oncogenic, depending on cellular context 4. Recently, considerable *NOTCH* gene mutations were identified in different types of squamous cell carcinoma 5, 6, 7, 8, 9, questioning the general role of Notch pathway in SCCs.

In this review, evidences highlighting the role of Notch in different SCCs are summarized. In particular, we explored the *NOTCH1* mutation patterns in different SCCs, analyzed their functional consequence based on current structural model of the Notch receptor. We suggest that most of the *NOTCH1* gene alterations are loss‐of‐function mutations. We discuss the intriguing possibility that a tumor suppressor role of Notch may be appreciated across diverse SCCs. The insights gathered from separate SCCs may have complementary advantages toward clearer understanding of Notch's role involved in squamous cell carcinogenesis.

## Notch Signaling

Notch signaling is a highly conserved signaling pathway in metazoan. It plays essential roles in multiple stages of metazoan development and tissue renewal. Its dysregulation is participated in a number of human diseases such as cancers and developmental disorders 10, 11.

NOTCH receptors are type I single‐pass membrane proteins. From *Drosophila* to mammals, the Notch homologues share highly conserved domain architecture 3. The large Notch extracellular domain (NECD) comprises 11–36 tandemly repeated epidermal growth factor‐like (EGF) repeats followed by a negative regulatory region (NRR) composed of three Lin12‐Notch repeats (LNR) and a hetero‐dimerization domain (HD). The HD domain is cleaved at S1 site by a furin‐like protease within the secretory pathway 12, generating a heterodimer that remains in a resting state, with its NRR domain being resistant to further protease cleavage 13, 14.

**Figure 1:**
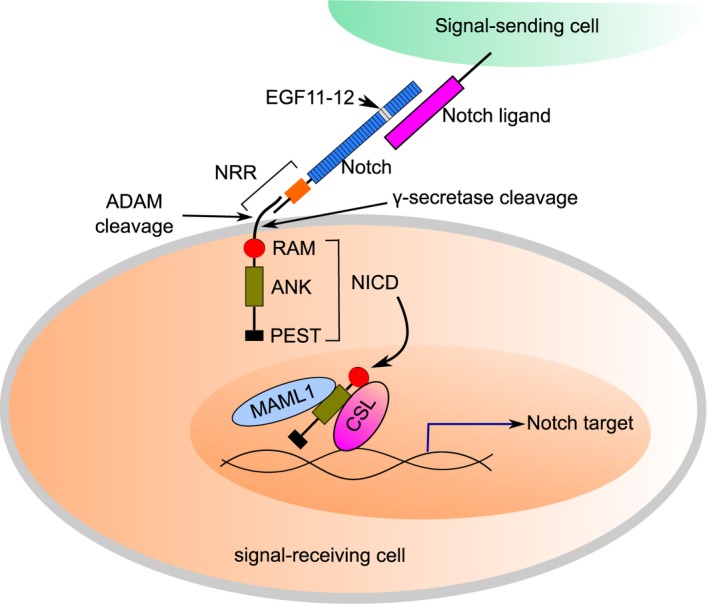
The canonical Notch signaling pathway. The interaction between Notch and its ligands from neighboring cells results in two successive cleavage event: cleavage at site S2 by ADAM protease and subsequent intramembranous cleavage at S3 by *γ*‐secretase complex. This ligand‐dependent activation process generates Notch intracellular domain (NICD). In the nucleus, NICD forms the transcription activation complex with transcription factor CSL and transcriptional coactivator such as MAML1, initiating the transcription of target genes.

The activation of Notch pathway usually involves the direct interaction between Notch and its ligands on cell surface (Fig. 1). Previous work demonstrated that EGF11 and EGF12 are the required elements for Notch1 to be able to recognize its ligands Jagged and DLL 15. Ligand binding leads to the cleavage at the juxtamembrane region (site S2) of Notch by ADAM (a disintegrin and metalloprotease) protease 16, 17. Shedding of the Notch extracellular domain facilitates subsequent intramembranous cleavage of Notch by *γ*‐secretase complex, generating Notch intracellular domain (NICD or ICN)^18^, ^19^. The NICD mainly contains membrane‐proximal RAM domain, an ANK domain, and C‐terminal PEST motif 3 **.** The NICD migrates to the nucleus and associates at high affinity with DNA‐binding factor CSL through the RAM domain 20. Meanwhile, the ANK domain weakly interacts with both CSL and a shorter sequence at the N‐terminal end of mastermind‐like 1 (MAML1) protein (the transcriptional co‐activator)^21^, ^22^. The formed transcription activation ternary complex NICD/CSL/MAML1 further upregulates the target genes (such as HES/HEY transcriptional repressors 23), which is known as the canonical Notch signaling pathway 3.

## Notch Signaling in Cutaneous Squamous Cell Carcinoma (CSCC)

Prevalent evidences support the notion that Notch activation can be growth repressive and differentiation inducing in CSCC. However, the detailed molecular mechanism underlying these effects remains not to be clearly elucidated.

### Genomic characterization of *NOTCH* in CSCC

Whole‐exome or *NOTCH1*/*NOTCH2* exons sequencing of primary CSCC or CSCC cell lines identified *NOTCH1* or *NOTCH2* mutations in ~75% of samples tested 5. The missense mutations were found to be localized to NECD EGF‐like repeats, NECD HD domain, and the intracellular RAM domain. In particular, selected missense mutations were further characterized to be loss‐of‐function mutations. Specifically, D469G from EGF‐like repeat domain and R1594Q from the NECD LNR‐C domain significantly reduced ligand‐mediated NOTCH1 activation, whereas P1770S from the RAM domain seems to interfere with Notch signaling at the level of transcription complex assembly. Furthermore, high‐frequent mutation of *NOTCH* in CSCC was confirmed by exome or targeted sequencing of CSCCs or squamoproliferative lesions 24, 25.

### Reduced notch levels in CSCC

Besides mutations, reduced NOTCH levels or activation observed in CSCC also support its tumor suppressor role. Decreased levels of NOTCH transcript and protein together with a parallel reduction in *HES1* gene (a common target of Notch pathway) were observed in CSCC cell lines 26. Furthermore, reduced levels of activated NOTCH1 were detected in CSCC, correlating with their *NOTCH1* mutation status 24. Protein and transcript levels of NOTCH1 were also found to be decreased in human CSCCs compared with nonlesional epidermis 27.

### Effects of *NOTCH* loss of function in CSCC

The functional consequences of attenuated Notch signaling in CSCC or keratinocyte (KC) were well demonstrated by several studies on loss of function of NOTCH1 receptor. Inhibition of Notch activation in primary keratinocytes by the expression of a dominant negative peptide competing for MAML1 binding to Notch/CSL complex can cause aggressive SCC formation depending on activated *ras* 26. Consistently, conditional transgenic mice were generated by expressing a dominant negative MAML1 protein to inhibit Notch signaling in the epidermis. These mice exhibited epidermal hyperplasia and developed spontaneous CSCC 28. Furthermore, the keratinocyte‐specific ablation of *NOTCH1* induced significant proliferation of the basal epidermal layer and deregulated expressions of multiple differentiation markers, indicating disrupted balance between growth and differentiation 29. In a later research using similar conditional gene deletion technology, effects of long‐term NOTCH1 deficiency were further characterized 30. The induced *Notch1* ^‐/‐^ adult mice showed epidermal hyperplasia followed by formation of skin tumors.

More functional insights were gained from functional or clinical observations utilizing Notch signaling inhibitors. In vivo treatment of grafted mice with *γ*‐secretase inhibitor DAPT (a pharmacological inhibitor of Notch activation) resulted in tumor‐promoting effects, depending on oncogenic *ras* 26. A recent clinical Phase III trial of Semagacestat, a *γ*‐secretase/Notch inhibitor, was halted in part because of an increased risk of skin cancers compared with those in the placebo arm 31.

### Molecular mechanism of notch in CSCC

**Figure 2:**
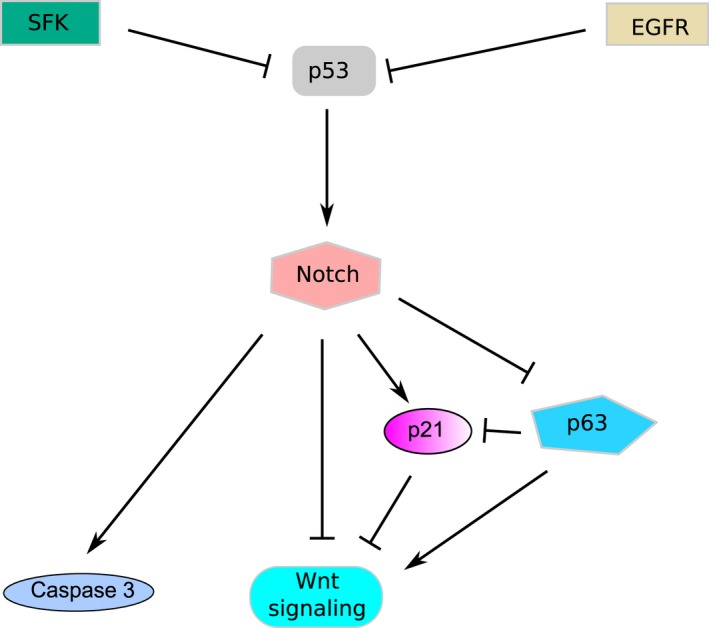
Tumor suppressive Notch signaling involved in the tumorigenesis of CSCC. Inactivation of Notch signaling contributes to CSCC. SFK, Src family tyrosine kinases; EGFR, Epidermal growth factor receptor. The signaling pathway components and their connections are described in detail in section 2.4 ‘Molecular mechanism of Notch in CSCC’.

Compared with other SCC types, the Notch‐related molecular mechanism for CSCC is relatively well characterized (Fig. 2). The disruption of canonical Notch/CSL/MAML‐mediated signaling pathways in transgenic mice by expressing epidermal DNMAML1 (a pan‐Notch inhibitor) resulted in hyperplastic epidermis and spontaneously developed CSCC 28. In mouse keratinocytes, cyclin‐dependent kinase inhibitor p21 was identified as a downstream positive target of NOTCH1, where activated NOTCH1 induces p21 expression to suppress growth in a CSL‐dependent manner 29, 30. p21's role in tumor suppression was earlier investigated where *p21* knockout keratinocytes exhibited significant downmodulation of differentiation markers and dramatic increase in proliferative potential 32. The inhibition of Notch signaling by genetic methods or by using pharmacological Notch inhibitors in human keratinocytes, together with activated *ras,* can cause aggressive SCC formation in grafted mice 26. This is similar to that previously revealed for ras‐transformed *p21* knockout keratinocytes 32, suggesting that NOTCH1 and p21 may overlap to perform their tumor suppressing functions. However, the general role of p21 remains to be investigated as p21 levels were selectively decreased in a subset of human skin SCCs tested 26, whereas in human keratinocytes, activation of NOTCH1 was found to lead to modest increase in p21 levels 33.

Notch1's tumor suppressor role in CSCC may involve beta‐catenin/Wnt pathway. In the epidermis of induced *Notch1* ^*‐/‐*^ mice, significantly high levels of free (unphosphorylated) beta‐catenin were observed 30. Loss of Notch1 signaling seems to activate beta‐catenin signaling in human CSCC 28, which can be repressed by the introduction of a dominant active form of NOTCH1^30^. In particular, p21 (a downstream mediator of Notch1‐induced growth arrest as we described above) also contributes to the downregulation of Wnt signaling, which likely account for the decreased levels of beta‐catenin activation by activated NOTCH1^34^. Furthermore, a negative feedback loop involving p63 is thought to modulate this pathway in keratinocytes. p63 is essential for maintaining the proliferative potential in epidermal stem cells 33, 35. NOTCH1 was shown to downmodulate p63 expression. On the other hand, p63 appears to function as a direct repressor of several Notch‐modulated genes as p63 knockdown led to substantially increased p21 expression and reduced Wnt signaling 33.

Caspase 3, involved in programmed cell death, was identified as another downstream mediator of NOTCH. Lack of caspase 3 resulted in increased proliferation and decreased differentiation of embryonic keratinocytes 36. However, the possible involvement of this pathway in CSCC remains to be clarified, although increased expression of survivin, an apoptosis inhibitor targeting caspase 3 and caspase 7, was observed in CSCC tumors 37, 38, 39.

p53 was characterized as a upstream positive regulator of NOTCH1. p53 is also an established cancer gene in CSCC 40, 41, 42 and HNSCC 43. Although p53 levels in cutaneous SCC are less characterized, somatic mutations of *TP53* are frequently found in many typical tumors including CSCCs, which is then likely to compromise p53 function 44. In keratinocytes, p53 was proposed as a positive regulator of Notch signaling. Specifically, p53‐responsive element has been identified in the Notch1 promoter 26, 45, 46. Consequently, NOTCH1 expression is upregulated by increased p53 in human primary keratinocytes and CSCC cell lines 26, 45.

A further connection exists between the p53‐Notch1 pathway and Src family tyrosine kinases (SFK) pathway. SFK are known oncogenes 47 and can be downregulated by Srcasm 48. Increased activation of SFK and decreased Srcasm levels were reported in CSCC compared with unremarkable epidermis 27, 49, 50. It was later reported that the transgenic mice with elevated SFK activity spontaneously formed CSCC, and cutaneous neoplasia can be markedly inhibited by increasing Srcasm levels 27. This intriguing study showed that increased SFK activity decreases the transcript and protein levels of p53 and NOTCH1, thus suggesting another mechanism to control NOTCH1 function.

EGFR was identified as another upstream mechanism controlling NOTCH1 function. EGFR is a well‐known determinant of epithelial cell proliferation. It is frequently overexpressed in epithelial tumors 43, 51 and persistently activated in keratinocyte tumors 52. In primary human keratinocytes, EGFR signaling suppresses differentiation and enhances proliferation by negatively modulating NOTCH1 transcript and protein levels through p53 41 in a similar way to SFK, suggesting Notch's central role in CSCC development.

## Notch Signaling in Head and Neck Squamous Cell Carcinoma (HNSCC)

The molecular pathogenesis of HNSCC has not been clarified yet 43. However, accumulating evidence may suggest the crucial role of Notch pathway in HNSCC, possibly in a similar manner to that in CSCC.

### Genomic characterization of *NOTCH* in HNSCC

In 2011, two independent studies about mutational landscape of HNSCC revealed that *NOTCH* is frequently mutated in HNSCC 6, 7. By whole‐exome sequencing of 74 HNSCC tumor‐normal pairs 6, mutations of *NOTCH1*,* NOTCH2*, and *NOTCH3* were identified in 14%, 5%, and 4% of HNSCC samples. In an independent exome‐sequencing study of 32 HNSCC primary tumors 7, *NOTCH1* somatic mutations were identified in 15% of patients, and following *TP53, NOTCH1* was identified as the second most frequently mutated gene. Consistently, early this year, a comprehensive genomic characterization of 279 HNSCCs 53 also revealed frequent *NOTCH1* mutations (19%). Among the identified mutations, considerable nonsense or splice‐site mutations were revealed which may have generated truncation sequences lacking the critical regions, while a bunch of missense mutations clustered in the NECD ligand‐binding region 6, 7, suggesting a tumor suppressive role for NOTCH in HNSCC.

### Genomic characterization of *NOTCH* in esophageal squamous cell carcinoma (ESCC)

HNSCC patients often develop second ESCC. HNSCC and ESCC have some common risk factors and may be closely related 54, 55. Similar mutation pattern of *NOTCH1* was also revealed by genomic characterization ESCCs 8. Exomic sequencing of 12 ESCCs in parallel with 12 esophageal adenocarcinomas (EAC) revealed frequent *NOTCH1* mutations in 21% of ESCCs but not in EACs 8. Notably, different *NOTCH1* alternation patterns were observed in patients from different ethnical populations: *NOTCH1* appears to mutate more frequently in North American ESCCs (21%) than Chinese ESCCs (2%). However, this concept was questioned by a later genetic landscape of ESCC samples of Chinese ancestry, which identified a high *NOTCH* (*NOTCH1‐3*) alteration frequency (13%) in Chinese cases 56.

### Functional characterization of notch pathway in HNSCC/ESCC

It was reported that *NOTCH1* was downregulated specifically in squamous neoplasms of oral mucosa and esophagus 57 and was variably reduced in primary oral SCC (OSCC) tissues 58. In another study, mRNA levels of *HEY1* (a target gene of Notch/CSL/MAML‐mediated signaling pathway) were found to be underexpressed in a subset (26%) of ESCC sample, which was significantly correlated with tumor depth of invasion 59.

The functional consequences of Notch levels were also investigated. Activation of Notch signaling by expressing full‐length NOTCH1 or its activated form ICN in human OSCC or HNSCC cell lines leads to cell growth arrest 9, 27, 60. Consistently, inhibition of NOTCH activation promoted tumor growth in a HNSCC cell line harboring wild‐type *NOTCH1[* 27], whereas activation of NOTCH1 resulted in reduced tumorigenicity in a mouse cancer model 9, 27 . Similarly, it was reported that Notch inhibition can promote transdifferentiation of normal esophageal squamous epithelial cells toward a BE (Barrett's esophagus, a precursor of esophageal adenocarcinoma)‐like metaplasia 61.

Although limited, accumulating evidences related to HNSCC/ESCC are consistent with Notch‐related molecular mechanisms for CSCC (Fig. 2). p21, p53, EGFR, caspase 3, and beta‐catenin/Wnt pathway related to Notch may also be involved in generation of HNSCC/ESCC. Activation of Notch signaling by stably expressing exogenous NICD in human OSCC cell line can lead to growth arrest, accompanied by reduced *β*‐catenin expression and dramatic increased *p21* and p53^60^. EGFR is overexpressed or activated in premalignant ESCC lesions 62 and is correlated with poor prognosis in HNSCC patients 63, 64, 65, 66, 67. Notch inhibition by DNMAML1 promotes invasive growth of transformed esophageal epithelial cells with EGFR overexpression and p53 dysfunction 68. Moreover, TP53 was frequently mutated in HNSCC cases 6, 7, 53, and the inactivation of p53 plays important role in HNSCC tumorigenesis 43, 69, 70. But it has not been clarified yet whether Notch signaling is closely involved in this.

Notably, apoptosis dysregulation also seems associated with ESCC/OSCC. Expression of survivin, an apoptosis inhibitor targeting caspase 3 and caspase 7, was found elevated in OSCC tissue 71, 72, whereas caspase 3 expression level is decreased 72. Consistently, caspase 3 is found associated with a favorable prognosis for primary resected ESCC patients 73, whereas survivin expression was proposed as negative prognostic factor of OSCC 74. However, different roles of caspase 3 in OSCC are also suggested. Pycnogenol can inhibit neoplastic cell transformation of HSC‐3 human OSCC cell, dependent on apoptosis with elevated caspase 3 activity 75. Increased caspase 3 expression is found in OSCC compared with normal oral epithelium 76. These findings question the apoptotic role of caspase 3, suggesting its nonapoptotic functions in oral cancers, as implicated in other cancers 77, 78, 79.

### Evidences suggesting *NOTCH* as oncogene in HNSCC

However, there are still considerable evidences questioning the tumor suppressor role of NOTCH1 in HNSCC. The main concern is regarding the elevated levels of Notch signaling pathway genes in different HNSCC/ESCC. Notch signaling pathway components (including Notch receptors, ligands, or targets genes such as *HES1*,* HEY1*, or *HEY2*) were found to be amplified or overexpressed in a subset of tongue tumor samples 80, in HNSCC tumors over normal mucosa 81, in OSCC 82, 83, 84, and frequently in ESCC samples 85.

However, the functional consequences of Notch pathway upregulation have been less characterized. Decreased NOTCH1 was associated with the inhibition of cell proliferation in the OSCC cell line 84. Blocking Notch activation by gamma‐secretase inhibitor can suppress OSCC growth in vitro 83 and inhibit the growth of human tongue carcinoma cell line 86. In addition, NOTCH1 was also proposed as a poor survival marker in ESCC 87.

## Notch Signaling in other Squamous Cell Carcinoma

As compared to HNSCC and CSCC, the role of Notch signaling in other types of squamous cell carcinoma is largely unclear. Evidences are gathered mainly from the sequencing research of lung SCC. A comprehensive genomic landscape of lung SCC was achieved by whole‐genome or mRNA sequencing of total 178 lung SCC samples. This identified *NOTCH1* as one of the significantly mutated genes, occurring in 8% of the testing samples. Most of the *NOTCH1* alterations (8 in 17) were truncating mutations, likely causing loss of function 88. Similarly, an analysis of publicly available lung SCC exome‐sequencing data revealed mutations of *NOTCH1* and *NOTCH2* at a combined frequency of 12.5% 5.

## A Complete View of *NOTCH1* Mutations Across SCCs

### Overall *NOTCH1* mutation patterns across SCCs

**Figure 3:**
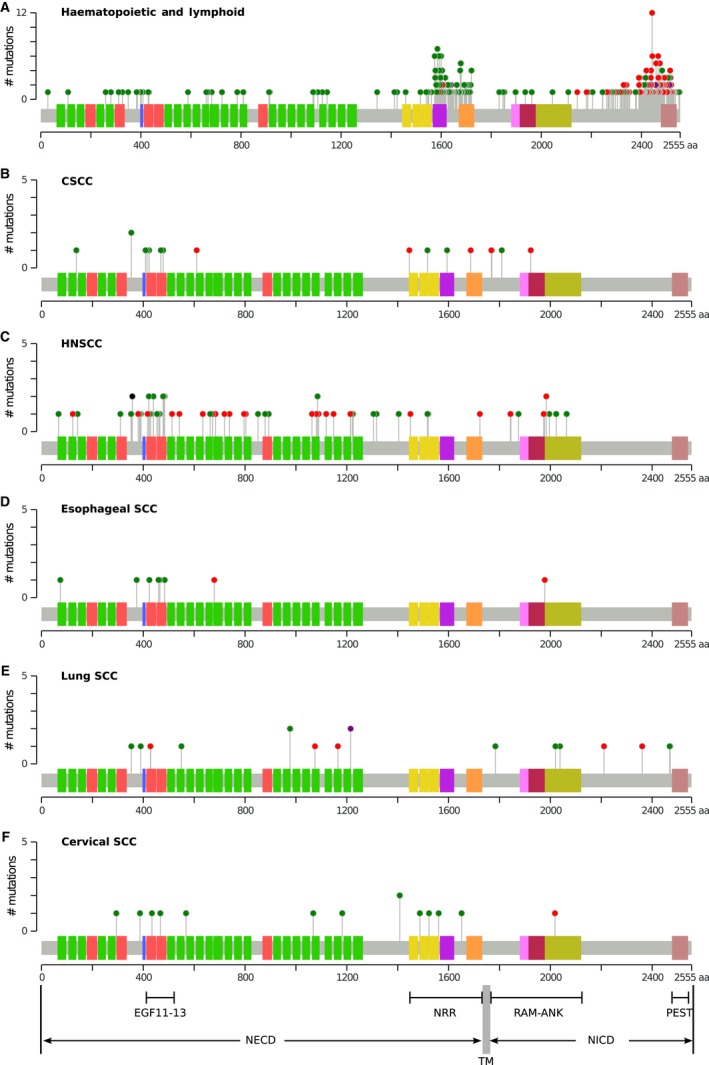
Mutation pattern across diverse SCCs. (A) *NOTCH1* mutations identified in tumors of hematopoietic and lymphoid where Notch1 is known as oncogene. Gene alteration data are obtained from COSMIC (B) *NOTCH1* mutations identified in CSCC 5. (C‐F) *NOTCH1* mutations identified in HNSCC, esophageal SCC, lung SCC, and cervical SCC, respectively. Gene alteration data are obtained from cBioPortal. In (A) and (B), the mutation data are mapped onto Notch1 with cBioPortal tool MutationMapper. The domain organization of Notch1 is labeled under panel (F). Green circles, missense mutations; red circles, nonsense or frame‐shift mutations. NECD, Notch extracellular domain; NICD, Notch intracellular domain; NRR, negative regulatory region; RAM, RBPj*κ* association module; ANK, ankyrin repeats; PEST, proline/glutamic acid/serine/threonine‐rich motif.

To gain insights of Notch alteration across diverse SCCs, we summarized publicly available *NOTCH1* mutation data in five different SCCs (Fig. 3B‐F), and analyzed the *NOTCH1* mutation features among them. Considerable nonsense or frame‐shift mutations occur within or before intracellular RAM‐ANK domain for transcription regulation, which is essential for Notch signaling (Fig. 1). Therefore, these early terminations likely result in inactive truncations. In addition, large number of missense mutations was also identified, but they cluster within the NECD region with 36 tandem EGFs, with a few in the NRR domain or RAM‐ANK domain.

This is in contrast to the *NOTCH1* alteration pattern revealed in hematopoietic malignancies, where Notch is known as tumor suppressor 4, 89. The majority of these mutations include missense mutations in the NRR domain and nonsense/frame‐shift ones in the PEST domain (Fig. 3A). Missense mutations in NRR domain likely destabilize inhibitor state of NRR domain, allowing ligand‐independent Notch activation 90. PEST domain is involved in proteasomal degradation of NOTCH 91, 92, and truncations lacking PEST region likely impair the degradation of NOTCH and augment Notch activation.

### Functional consequences of *NOTCH1* missense mutations across SCCs

More Compelling evidences for Notch loss of function across SCCs may result from the functional consequences of the considerable missense mutations. To our knowledge, only three mutations identified from CSCC have been characterized 5. To gain more insights into these mutations, we resorted to the accumulating structural knowledge of Notch signaling.

#### Mutations in the RAM‐ANK domain

**Figure 4:**
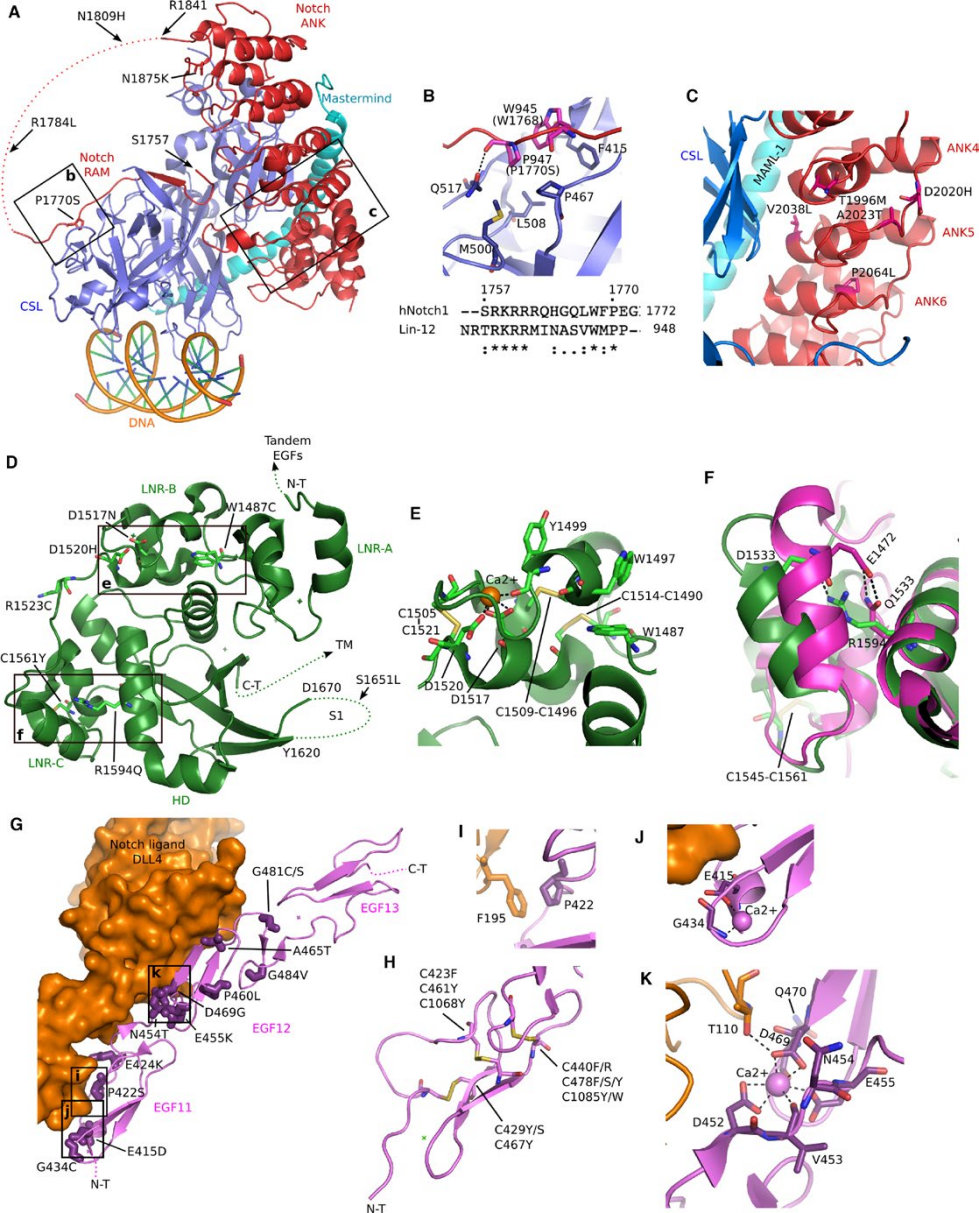
Up‐to‐date structural information of Notch signaling predicts the deleterious consequences of missense mutations identified in multiple SCCs. (A) Ribbon diagrams of the *C. elegans* transcription activation complex of CSL (blue), Notch RAM‐ANK (red), N‐terminal domain of Mastermind (cyan), and DNA (orange) (pdb id: 2FO1). Mutations in RAM domain of *Human* NOTCH1 identified in SCCs were mapped onto this complex as blue sticks. (B) A close‐up view of the marked region in (**A**) shows the functional consequence of mutation P1770S. *Human* NOTCH1 P1770 and W1768, counterparts of worm Lin‐12 P947 and W945, are labeled in bracket. (C) Mutations in ANK domain of *Human* NOTCH1 are mapped onto the *Human* complex of CSL (blue), NOTCH1 ANK (red), N‐terminal domain of MAML1 (red), and DNA (orange) (pdb id: 2FX8). This zoomed view corresponds to the marked area in (A). (D) Mutations in NRR domain of *Human* NOTCH1 are mapped onto the structure of *Human* NOTCH1 NRR domain (dark green, pdb id: 3ETO). (E) A zoomed view of the marked region in (D). (F) A close‐up view of the marked region in (D). The aligned *Human* NOTCH3 NRR domain structure (pdb id: 4ZLP) is shown in magenta. (G) Mutations at noncysteine positions of *Human* NOTCH1 ligand‐binding region (EGF11‐13) are mapped onto the *Rat* NOTCH1 EGF11‐13/DLL4 complex structure (pdb id: 4XLW). (H) Due to the conserved positions of disulfide bonds in EGF module, mutations at cysteine positions of *Human* Notch ligand‐binding region (EGF11‐13) and another region (EGF28) are mapped onto the EGF 11 from (A) to show their relative positions in EGF module. (I‐K) Zoomed views of the marked regions in (G).

Analysis of the *Caenorhabditis elegans* (*C. elegans*) Lin‐12 RAM‐ANK/CSL/Mastermind/DNA complex structure 93 (Fig. 4A) shows that P1770 in RAM domain (conserved between human and worm, Fig. 4B) inserts into a hydrophobic pocket of the transcription factor CSL, thereby P1770S is predicted to reduce the binding of NICD to transcription factor. This is consistent with the previously reported results 5. R1784L and R1809H mutations are located on the flexible loop, and thus are likely to be benign (Fig. 4A). With regards to mutations in ANK domain, mapping them onto ANK/CSL/MAML‐1/DNA complex structure revealed that they cluster inside the ANK4‐6 motifs (Fig. 4C). V2038L and T1996M mutations face bound CSL, and therefore likely interfere with the binding of transcription factor. A2023T, D2020H, and P2064L mutations are predicted to disrupt structure of ANK4‐6 motifs that are involved in CSL binding, and therefore likely to be deleterious.

#### Mutations in the NRR domain

Several mutations were found in the NOTCH1 NRR region. Mapping these mutations on the *Human* NOTCH1 NRR crystal structure showed that they cluster in the LNR‐B and LNR‐C domain (Fig. 4D). R1523C may cause cysteine crosslink, impairing Notch function. D1517 and D1520 together with other residues form a Ca^2+^‐binding site (Fig. 4E), which is necessary for the stabilization of LNR‐B. LNR‐B is also stabilized by hydrophobic stacking between W1487 and W1497. Moreover, there are three disulfide bonds around this region. Thus, the mutations in this region (W1487C, D1517N, and D1520H) may destabilize LNR‐B, likely interfering with the correct folding of functional Notch1 molecule.

In LNR‐C domain, C1561 forms disulfide bond with C1545 (Fig. 4F), thus C1561Y likely destabilize the structure and the unpaired and solvent‐exposed C1545 then probably leads to cysteine crosslink between molecules. In addition, the R1594Q was previously characterized and shown to be a reduced ligand‐mediated activation of NOTCH1^5^. A very recent structural study of *Human* NOTCH3 NRR domain may provide a basis to understand the effect of a glutamine at this position 14: NOTCH3 has Q1533 at this position and the shorter side chain allows the LNR‐C domain to pack more tightly on HD domain (Fig. 4F), likely stabilizing the inhibitory state and decreasing the level of Notch activation.

#### Mutations in the ligand‐binding region

EGF11‐12 region of NOTCH1 is the required elements for Notch to be able to recognize its ligands Jagged and DLL 15. Quite a few (22 in total) NECD missense mutations occur in this small (77 residues) region. In contrast, there are no mutations within the neighboring EGF13 that is not involved in ligand binding (Fig. 3). Recent structure of Rat NOTCH1 EGF11‐13 in complex with its ligand DLL4^94^ provides a basis to analyze the functional consequence of mutations at this region. Surprisingly, frequent mutations (10 out of 22) occur at cysteine positions (Table 1 and Fig. 4H). The disulfide bonds at these cysteine sites are necessary to keep the conserved EGF module structure. Thus, these mutations are likely to cause misfolding of NOTCH1 to affect Notch function. Similarly, we noticed that all the missense mutations on EGF28 occur at cysteine sites and they probably disrupt Notch1 folding likewise (Table 1 and Fig. 4H). Moreover, the localizations of mutations at noncysteine sites surprisingly showed that they cluster near the DLL4 ligand‐binding interface of NOTCH1 EGF11 and EGF12 (Fig. 4G), and therefore are predicted to affect Notch ligand binding. G434C and G481C are solvent exposed, so they may cause cysteine crosslink to affect Notch function. In particular, P422 (Fig. 4I and D469 (Fig. 4k) mutations are directly involved in ligand binding. This can explain the previous finding that D469G showed reduced ligand‐mediated activation 5. In addition, E415 and G434 participate to form a calcium‐binding site in EGF11 (Fig. 4J). D469 and E455 cooperate with residues D452, V453, and D470 to form another calcium‐binding site in EGF12, with N454 located between them (Fig. 4k). A previous mutagenesis study revealed that this calcium‐binding site is essential for Notch ligand binding 95. Therefore, the E415D, G434C, D469G, N454T, and E455K are predicted to affect ligand‐mediated activation of Notch through the disruption of calcium‐binding site.

**Table 1:** Compilation of *NOTCH1* mutations implicated in diverse SCCs and their predicted functional consequences

Notch1 mutations	Positions on Notch1	Predicted functional consequences	Associated SCC type
	Ligand‐binding region		
E415D	EGF11	Disrupting calcium binding or ligand binding	Cutaneous
P422S	EGF11	Disrupting ligand binding	Head & neck
C423F	EGF11	Disrupting Notch folding or causing cysteine crosslink	Cutaneous
E424K	EGF11	Disrupting ligand binding	Esophagus, Head & neck
C429Y	EGF11	Disrupting Notch folding or causing cysteine crosslink	Head & neck
C429S	EGF11	Disrupting Notch folding or causing cysteine crosslink	Lung
G434C	EGF11	Disrupting calcium binding or causing cysteine crosslink	Cervical
C440F/R	EGF11	Disrupting Notch folding or causing cysteine crosslink	Head & neck
N454T	EGF12	Disrupting calcium binding	Head & neck
E455K	EGF12	Disrupting calcium binding	Head & neck
P460L	EGF12	Substituting conserved residue of EGF module	Esophagus
C461Y	EGF12	Disrupting Notch folding or causing cysteine crosslink	Head & neck
A465T	EGF12	Disrupting ligand binding	Esophagus, Head & neck
C467Y	EGF12	Disrupting Notch folding or causing cysteine crosslink	Cervical
D469G	EGF12	Disrupting ligand binding or calcium binding	Cutaneous
C478F	EGF12	Disrupting Notch folding or causing cysteine crosslink	Cutaneous
C478S/Y	EGF12	Disrupting Notch folding or causing cysteine crosslink	Head & neck
G481S	EGF12	Benign	Head & neck
G481C	EGF12	Causing cysteine crosslink	Head & neck
G484V	EGF12	Benign	Esophagus, Head & neck
C1068Y	EGF28	Disrupting Notch folding or causing cysteine crosslink	Cervical
C1085Y/W	EGF28	Disrupting Notch folding or causing cysteine crosslink	Head & neck
	NRR		
W1487C	LNR‐B	Disrupting Notch folding or causing cysteine crosslink	Cervical
D1517N	LNR‐B	Disrupting calcium binding or Notch folding	Cutaneous, Head & neck
R1520H	LNR‐B	Disrupting calcium binding or Notch folding	Head & neck
R1523C	LNR‐B	Causing cysteine crosslink	Cervical
C1561Y	LNR‐C	Disrupting Notch folding or causing cysteine crosslink	Cervical
R1594Q	LNR‐C	Stabilizing the inhibitory state of NRR	Cutaneous
S1651L	HD	Benign	Cervical
	RAM‐ANK		
P1770S	RAM	Decreasing transcription factor binding;	Cutaneous
R1784L	RAM	Benign	Lung
N1809H	RAM	Benign	Cutaneous
N1875K	ANK	Benign	Head & neck
T1996M	ANK	Interfering with transcription factor binding	Head & neck
D2020H	ANK	Disrupting Notch folding or changing ANK conformation	Lung
A2023T	ANK	Disrupting Notch folding or changing ANK conformation	Head & neck
V2038L	ANK	Interfering with transcription factor binding	Lung
P2064L	ANK	Disrupting Notch folding or changing ANK conformation	Head & neck

The missense mutants are from the analysis of Figure 3B‐F. Only mutations identified in ligand‐binding region EGF11‐13, EGF28, NRR domain, and RAM‐ANK region are listed and analyzed.

## Conclusions

The role of Notch pathway in tumorigenesis is highly variable. Specifically, it can be tumor suppressive or pro‐oncogenic, depending on the cellular context 4. As we have described, the tumor suppressor role of Notch was relatively well established in CSCC, unlike for HNSCC 43. Until recently, the *NOTCH1* has drawn much attention as it was identified as one of the most frequently mutated genes in SCCs of cutaneous 5, head and neck 6, 7, 53, esophageal 8, 56, and lungs 88. We summarized the *NOTCH1* mutation patterns in five different SCCs, and explored the biological consequences of these mutations based on the Up‐to‐date structural information of Notch signaling. Our analysis suggested that the Notch loss‐of‐function mutations occur across diverse SCCs. Consistently, considerable functional evidences obtained for HNSCC suggested substantially overlapped aberrant signaling events between HNSCC and CSCC, implicating that Notch may be tumor suppressive in HNSCC as that in CSCC. This concept may need to be further validated with the studies targeting SCCs from other tissues, such as lung and cervical whose molecular pathogenesis remains poorly understood. However, the oncogenic role of Notch in HNSCC is likely, as the increased levels of Notch signaling components in OSCC/ESCC were reported 82, 84, 85, although their biological consequences remain to be determined.

A tumor suppressor such as NOTCH is a possible, but challenging therapeutic target 96. Therefore, current treatments of SCC are directed toward upstream regulators and downstream effectors of the Notch pathway, which has been extensively studied and well reviewed for CSCC 97. New understanding of Notch signaling may lead to therapeutic development for less‐characterized SCC types. Notably, structure‐guided analysis of the functional consequences of NOTCH cancer mutations, as we present in this review, may provide new clues and advance the development of drugs that can restore aberrant Notch signaling. This strategy was proved to be applicable for another tumor suppressor, p53^98^, ^99^, and therefore may represent an attractive future direction of targeted therapy of SCC. Emerging new understanding of the Notch pathway in diverse SCC types will likely enhance the possibility for improvement in therapy of SCC.

Moreover, besides at the levels of mRNA and protein levels, Notch activation can be regulated at other different levels such as ligand binding, protease cleavage, or even post‐translational modifications. It may be fruitful to target this broader Notch pathway to gain a more panoramic understanding of the important role of Notch in SCCs. A nice example is a recent research on the tumor suppressor role of Notch pathway in bladder cancer, revealed by the characterization of multiple components of Notch pathway 100. We have recently characterized a Notch‐modifying enzyme Xxylt1 101 that is frequently amplified in specific cancer types lacking loss‐of‐function *XXYLT1* mutations. Surprisingly, of the six cancer types with highest *XXYLT1* amplification frequency, three are squamous cell carcinomas: SCCs of head and neck, lung, and cervical. Considering that Xxylt1 can negatively regulate Notch activation 102, this may suggest a novel pathway changes to comprise Notch activation in the tumorigenesis of these cancer types. Therefore, considering the complex roles of NOTCH in tumorigenesis, expanding our knowledge of Notch pathway will surely benefit our understanding of the complicated Notch in cancer.

## Conflicts of Interest

The authors declare that there are no conflicts of interest.
